# Progress in Pediatrics

**Published:** 1902-10-11

**Authors:** 


### PROGRESS IN PEDIATRICS.

**Chloroma.**—This is an affection chiefly of childhood and adolescence regarding which little was known in this country until the publication recently of two cases by Dr. Melville Dunlop[1] and Dr. Byrom Bramwell.[2] The disease has been variously described as green cancer, fibro-plastic growths, amorphous fibrous tumours, and pigmented myxo-sarcoma or chloromyxo-sarcoma. Dr. Dunlop's case was that of a boy, aged five years, who was admitted to hospital because of great weakness and swelling of the face. He was extremely pale, various ecchymoses had appeared about the body, and any scratch of the skin was followed by rapid suppuration. Exophthalmos, with swelling of the veins of the eyelids, deafness, and an attack of pyrexia with albuminuria had subsequently developed. Later swellings appeared on the hard palate, on the lower jaw, in each temporal region, in the eyelids, and on the eyeballs. The last-named commenced as a pale greenish-yellow tumour of a jelly-like consistence, which started at the inner canthus of the right eye and extended over the whole of the cornea. Examination of the blood showed a marked diminution of red corpuscles and an increase of leucocytes to 245,000, of which 73 per cent. were lymphocytes. The boy died from exhaustion. At the necropsy masses of green tissue were found growing from the periosteum and in the medulla of many of the bones, notably the sternum, the ribs, the skull, and the vertebræ. During the progress of the necropsy the green colour gradually faded from the parts exposed. Masses of green tissue were also found in the kidneys the mesenteric glands, the pancreas, the soft parts around the skull, and in the mouth. The green colour of these growths, which are lymphoid in character, has been much discussed, but Dr. Dunlop thinks it is probably due to some chemical product allied to lipochrome, which, on exposure to light, becomes oxidised and loses its colour. Virchow and others are agreed that the colouring matter lies in the adipose granulations which are found in and between the cells. Dr. Bramwell in his paper summed up the clinical features of chloroma as follows. 1. Lymphoid deposits in the orbits, temporal fossæ, and periosteum of the skull with resulting exophthalmos, hæmorrhages in the retinæ, optic neuritis, deafness, facial palsy, and nasal obstruction. 2. The green colour of the lymphoid deposits. 3. A profound blood change characterised by anæmia, cachexia, hæmorrhages into the skin, and bleeding from the mucous membranes. 4. Lymphoid deposits in the bone marrow, the spleen, the lymphatic glands, and other viscera. He discussed the question as to whether chloroma was a definite and distinct disease, or merely a variety of acute lymphatic anæmia.

**The Feeding of Infants.**—This subject continues to attract much attention and to produce much discussion. It is a curious fact that while in the various children's hospitals simplicity of diet is the thing aimed at, in private practice every new food and every elaborate method of preparing food seem at once to find numerous supporters both amongst the laity and the medical profession. It is impossible even to enumerate all the papers written on this subject, but we may refer those interested to the following. The whole subject of the modification of milk was fully treated by the greatest authority on this question, Dr. T. M. Rotch,[3] at the last meeting of the British Medical Association. Butter-milk is recommended by Dr. Adolf Baginsky[4] as an infant food, and more especially after an attack of diarrhœa. An address on patent foods by Dr. Robert Hutchison[5] is well worthy of study by those interested in the feeding of infants. In a coldly critical but scientifically accurate spirit he destroys many illusions cherished by the public and also possibly by some members of the medical profession. On the subject of fatty foods he considers the emulsions of cod-liver oil, of petroleum, and of the pancreas. For the first of these there is no necessity, because cream is as easily digested and absorbed, is more palatable, and is considerably cheaper. Petroleum emulsion is useless as a food, for it can never be absorbed, and every particle swallowed is discharged by the bowel. Pancreatic emulsion is composed of emulsified lard flavoured with clove oil, and is an elegant preparation which possesses no advantage over butter in the matter of digestibility or nutritive value. An important part of the lecture consisted in a complete analysis of all the patent infants' foods which are at present before the public, with a description of their preparation, and a criticism of their value in medical practice.

**Acute Pyelitis in Infancy.**—Some valuable information on the symptoms and treatment of this affection is supplied in a paper by Dr. John Thomson.[6] Undiagnosed it is an alarming illness, but under proper treatment it is a very curable one. The most striking symptom is a rigor or chill at the onset, which was present in four out of eight cases. A rigor is extremely rare in infancy, and its occurrence in pyelitis is therefore of great diagnostic value. The other symptoms are pyrexia (remittent in character), delirium, slight vomiting, marked restlessness, and distress. Symptoms of implication of the urinary tract may be slight and so indefinite that their significance is often overlooked. There may be screaming attacks from renal colic, or painful micturition, or soreness of the vulva (the affection usually attacks female infants), or tenderness on palpation of the kidney. Pus is present in the urine in considerable amount, and the bacillus coli communis abounds. He thinks the disease probably originates from the passage of this organism up the urethra to the kidney, or from the bowel into the blood stream. The essential part of the treatment consists in rendering the urine neutral by the administration of alkaline remedies, and keeping it so until all the symptoms have disappeared. Citrate of potash in full doses, 36 to 48 grs. in the 24 hours, secures this object, and the dose must then be gradually diminished so as to prevent too depressing a result. Dr. James Ritchie[7] has also recorded a case of this affection in a female infant aged seven and a-half months. She suffered from a series of rigors with remittent pyrexia. Relief and cure followed the use of citrate of potash.

1 Lancet, Feb. 15, 1902, p. 451. 2 Ibid., Feb. 22, 1902, p. 520. 3 Brit. Med. Jour., Sept. 6, 1902, p. 653. 4 Ibid., p. 692. 5 Lancet, July 5th, 1902, p. 1. 6 Scot. Med. and Surg. Jour., 1902, Vol. XI. No. 1, p. 7. 7 Ibid., p. 1.

*(To be continued.)*

